# Relationship among Nutritional Intake, Anxiety, and Menstrual Irregularity in Elite Rowers

**DOI:** 10.3390/nu13103436

**Published:** 2021-09-28

**Authors:** Mana Miyamoto, Yuko Hanatani, Kenichi Shibuya

**Affiliations:** 1 Graduate School of Health and Welfare, Niigata University of Health and Welfare, Niigata 950-3198, Japan; shibuya@nuhw.ac.jp; 2 Japan Rowing Association, 4-2 Kasumigaoka-machi, Shinjuku-ku, Tokyo 160-0013, Japan; yu-ko1027@nifty.com; 3 Department of Health and Welfare, Niigata University of Health and Welfare, Niigata 950-3198, Japan

**Keywords:** athletes, female, mental stress, amenorrhea

## Abstract

There is evidence showing that excessive mental stress is detrimental to the menstrual period, and it is known that many elite athletes are highly susceptible to mental anxiety. This study investigated the nutritional intake and mental anxiety of 104 relatively young elite endurance athletes aged 16 to 23 years and used a multiple logistic model to examine the factors that might be related to menstrual irregularity. Calcium intake was marginally associated with the occurrence of menstrual irregularities (odds ratio = 1.004, *p* = 0.030), whereas there were strong associations between body mass or state anxiety and menstrual irregularities in elite athletes (odds ratio = 0.557, *p* = 0.035 for body mass; odds ratio = 1.094, *p* = 0.006 for state anxiety). These results suggested that state anxiety would be an important factor causing menstrual irregularity in elite endurance athletes. It is recommended that elite athletes are monitored for anxiety levels and develop a strategy for stress management.

## 1. Introduction

A regular menstrual cycle is attributed to fertility and physical and mental well-being, whereas longer menstrual cycles are correlated with stress [1,2]. Intense training triggers the onset of the female athlete triad, which comprises the following interrelated characteristics: low energy availability (LEA), menstrual dysfunction, and low bone mass [2]. Energy availability (EA) is defined as (energy intake [EI; kcal] − energy expenditure from exercise [EEE] [kcal])/fat free mass (FFM) (kg). The recommended EA level for maintaining a healthy body is 45 kcal/kg FFM/day [3]. Chronic EA decreases below 30 kcal/kg FFM/day are associated with the impairment of various physical functions (e.g., functional hypothalamic amenorrhea, estradiol suppression, and progesterone suppression) [4]. LEA can be caused by inadequate EI, high total energy expenditure, or a combination of the two. It might also be associated with disordered eating, incorrect or overly rapid weight loss programs, or a failure to meet energy requirements during periods of heavy training or competition [3]. There are many cases of menstrual disorders and fatigue fractures due to reduced EA levels in female athletes [2,5,6,7,8]. Amenorrhea, or exercise-related menstrual irregularities, is a unique problem in women and in female athletes. Resistance training and energy restriction are associated with an increase in estradiol and beta-endorphin, which reduces gonadotropin-releasing hormone and luteinizing hormone (LH) pulsatility leading to menstrual disruption [9,10].

There are a limited number of studies examining the effects of mental anxiety on menstrual cycle characteristics and menstrual disorders in female athletes. In particular, the relationship between anxiety and menstrual disorders in top-level female athletes who compete in world-class competitions, including the Olympics, has not been thoroughly studied. Elite female athletes are likely to be subjected to a great deal of mental anxiety due to the high intensity of their daily training and the many opportunities they have to participate in major national and international competitions [11]. It was known that many individuals with high scores of 40 or more on state anxiety by the State and Trait Anxiety Inventory (STAI) were associated with mood and depression disorders [12,13]. Then, it was reported that there was a relationship between abnormal eating behavior, such as anorexia, and depression or mood disorders [14]. On the other hand, increased anxiety might activate the hypothalamic-pituitary-adrenal (HPA) system, inhibit gonadotropin-releasing hormone (GnRH) secretion, and delay or suppress LH secretion [15,16]. In addition, abnormal LH secretion can cause menstrual irregularities and amenorrhea [17,18]. Therefore, it is important to know the extent to which anxiety affects the occurrence of menstrual irregularities in top female athletes who are under greater anxiety in order to protect their normal reproductive function and physical and mental health. In this study, the effects of nutritional status and anxiety on menstrual function in elite rowing athletes was examined.

## 2. Materials and Methods

### 2.1. Participants

The respondents were 104 elite female athletes from a Japanese rowing team aged 16–23 years who were all preparing for international competitions (World Junior Rowing Championship and World U23 Rowing Championship). All Japan national team rowers who gave their consent participated in the present study. As a result, all national team members in the U19 and U23 category were included in the study. The data from each rower were investigated from April 2018 to October 2020. Their exercise training programs were developed in collaboration with the rowing coaches of the Japan National Rowing Team. A preliminary power analysis indicated that a sample size of 101 would provide 80% power using multiple logistic analysis with a level of significance of 0.05. The Ethics Committee of Niigata University of Health and Welfare provided approval to carry out the study (Approval #17982-180606). After they had received a full explanation of the nature of the study procedure and its noninvasiveness, each participant provided written informed consent.

### 2.2. Dietary Intake, Body Mass, and Body Composition

The subjects tracked their dietary intake using a meal-recording method. Athletes sent a photo of each meal (breakfast, lunch, dinner, and snacks) for 3 days (one non-training day and two training days) during 1 week to a national team dietitian. The dietitian analyzed the intake of each nutrient from these photos. Nutrient information was obtained from the Japan National Nutrient Database or product-specific nutrition facts panels. Daily nutrient intake information (total energy intake (kcal), macronutrients (g and %), and fiber (g)) was averaged. The body mass (BM) and body fat percentage (%BF) were measured on a commercially available home scale (BC-314, Tanita. Co.,Tokyo, Japan), for which validity was confirmed in a previous study [19]. Body mass index (BMI) was calculated as weight (kg)/height (m^2^). Height, BM, and %BF were measured only once, at the time of the investigation.

### 2.3. Energy Availability

EA was calculated from the dietary intake records, exercise training records, and estimated FFMs according to the following formula: energy intake (kcal] − EEE (kcal))/FFM (kg) [3,4]. EEE was calculated using the average of maximal oxygen uptake and the heart rate during training and daily life using a heart rate monitor (Polar Vantage M, Polar Japan) according to previous studies [7,20]. The established threshold of 30 kcal/FFM kg/day was used as a reference level to compare the subjects’ EA to the level below which adverse health outcomes have been detected [3]. At the first evaluation, all participants were informed of the concept of energy availability, the required energy intake for female rowers, and the importance of maintaining a good nutritional status for their performance and menstrual cycle according to guidelines on nutrition support in athletes [3].

### 2.4. State and Trait Anxiety and Menstrual Cycle

State and trait anxiety were measured using the State and Trait Anxiety Inventory (STAI) [21]. The menstrual cycle was tracked (paper/pen calendar) and reported by the participants. State and trait anxiety were measured only once, at the time of the investigation.

### 2.5. Statistical Analyses

Data were assessed for normality using the Shapiro–Wilk test. All data had normality, except for the data on menstrual irregularities. Statistical analyses were conducted using MASS in R. [22], and the independent variables introduced into the multiple logistic models were determined based on Akaike’s Information Criterion (AIC). We then performed multiple logistic regression analysis to estimate the risk of menstrual irregularity, including nutritional intake, physical characteristics, state anxiety, and trait anxiety. The inclusion of variables in the models was based on existing knowledge of the risk factors for menstrual irregularity. After constructing models for the menstrual irregularity, nutritional intake, and mental anxiety based on multiple logistic regression analysis, the area under curve the receiver-operating characteristic (ROC) curve (AUC) of the model was calculated to evaluate the power of the model. Bonferroni correction for the number of variables of the multiple logistic models was used for the repetition of testing.

## 3. Results

### 3.1. Physical Characteristics

The mean %BF was 23.8 with a 95% confidence interval (CI) of 22.7–24.8 with 24 athletes having a %BF below 20% (Table 1). The mean BMI was 22.3 (95% CI of 22.1–22.5). No athletes had a BMI less than 18.5, whereas one athlete had a BMI below 20. This athlete had no menstrual irregularity. The mean state anxiety value was 37.8 (95% CI: 36.1–36.4) with 41 athletes exhibiting a state anxiety above the clinical cut-off of 40. 

### 3.2. Macronutrient Intake

The mean energy intake (kcal) was 2665.4 (95% CI: 2572.2–2578.7), and the mean EA was 37.1 (95% CI: 35.0–39.2). EA values less than 35, 40, and 45 were observed in 49, 66, and 85 athletes, respectively (Table 2). The mean protein intake (g) was 109.2 (95% CI: 104.8–113.7), whereas the mean protein intake per body mass (Pro/BM, g/kg) was 1.7 (95% CI: 1.6–1.8). Pro/BM values of 1.0 or above, above 1.5, and 2.0 were found in 103, 73, and 24 athletes, respectively. The mean carbohydrate (CHO) intake was 349.1 (95% CI: 334.5–363.6), whereas the mean CHO intake per body mass (CHO/BM, g/kg) was 5.5 (95% CI: 5.3–5.8). The numbers of athletes with a CHO/BM ratio > 5 and > 6 were 61 and 35, respectively.

### 3.3. Micronutrient Intake

The mean iron (Fe) intake (mg) was 12.4 (95% CI: 11.7–13.0) with 28, 82, and 95 athletes showing an Fe intake below 10, 15, and 18, respectively (Table 3). Meanwhile, the mean calcium (Ca) intake (mg) was 860.6 (95% CI: 793.3–927.9), with a Ca intake of more than 1000 and 1300 found in 36 and 11 athletes, respectively.

### 3.4. Multiple Logistic Analysis

The AUC of the ROC was 0.79 for the multiple logistic models. The independent variables to be introduced into the multiple logistic models were determined based on AIC. Significant factors related to menstrual irregularity, including Ca intake, body mass, and state anxiety, were identified (*p* = 0.030, *p* = 0.035, and *p* = 0.006, respectively) from the multiple logistic analysis (Table 4). The odds ratios for Ca intake, body mass, and state anxiety were 1.004, 0.557, and 1.094, respectively. 

## 4. Discussion

Using multiple logistic analysis, state anxiety and macronutrient intake, but not energy, were found to be strong factors in menstrual irregularities in elite endurance young female athletes. Although there is evidence of mental health problems affecting the menstrual cycle [17,23,24], few reports have shown a link between the mental anxiety imposed on elite athletes and the menstrual cycle. Forty-six percent of elite athletes surveyed in Australia had mental health problems, with 53.4% of those affected being females [14]. In this study, there was an average STAI score of 37.8 for state anxiety and 42.1 for trait anxiety amongst the athletes. Those scores were strongly correlated in the present study (*p* < 0.001). However, state anxiety was strongly associated with menstrual irregularity, but trait anxiety was not significantly associated with menstrual irregularity based on the multiple logistic analysis in the present study. Furthermore, of 104 athletes, 41 for state anxiety and 57 for trait anxiety scored more than 40 points, which is considered a clinical cut-off point [11]. This ratio was much higher than that of the general population (19.5–20.1%) [25], and it can be inferred that elite athletes are in a state of mental instability. This is the first report linking the relationship between mental anxiety and menstrual irregularity in elite athletes. A previous study using females who were not athletes suggested that a higher level of anxiety is associated with irregular menstrual cycles [26]. In addition, premenstrual syndrome might contribute to anxiety. Since these factors are closely related, further studies are needed to clarify their effects on menstrual irregularities.

Ca intake was a slightly significant factor for menstrual irregularity (odds ratio = 1.004, *p* = 0.030). Further research on the relationship between Ca intake and menstrual irregularities is needed. In addition, we found a significant and negative association between body mass and menstrual irregularity (odds ratio = 0.557, *p* = 0.035) in the present study. This result suggests that weight gain decreased the incidence of menstrual irregularities, which is consistent with results of previous studies [3].

It is suggested that mental health problems cause menstrual irregularities through activation of the HPA axis [15] leading to an inhibition of the requisite preovulatory LH surge and consequent suppression of ovulation observed with higher follicle-stimulating hormone levels [5,27,28]. The present study suggests that menstrual irregularities might occur in elite athletes under conditions of intense mental anxiety. None of the athletes had a BMI below 18.5, and one athlete with a BMI less than 20 did not have symptoms of menstrual irregularities. The energy intakes and the state/trait anxiety scores of the athlete with the lowest BMI were 3550 kcal/day and 37/30. However, our previous study reported that increased energy intake significantly reduces the occurrence of menstrual irregularities [29]. Anxiety could have altered her food intake, which might have affected her menstrual irregularities. In addition, mental stress might lead to anxiety, which might lead to changes in menstruation. Further studies are needed on the relationship among energy intake, mental stress/anxiety, and the occurrences of menstrual irregularities. This study is valuable since it is the first to suggest that an increase in state anxiety might be related to menstrual irregularities in athletes with inadequate energy intake but no apparent energy deficit in terms of BMI.

In the present study, we used STAI to score state anxiety. To clarify the relationship between mental anxiety and menstrual irregularities in more detail, other measures of mental stress should be used. If we could also measure the hormones that cause menstrual irregularities to clarify the physiological effects of mental stress/anxiety on menstrual irregularities, we might be able to more accurately understand menstrual cycles in athletes. Nevertheless, there have been no studies that have investigated the relationship between mental anxiety and menstrual irregularities in elite athletes, and the results of the present study could contribute to future development in this research field. In the future, longitudinal studies will help to interpret the results of the present study correctly. Further research is thus needed. With the collection of more large-scale data, we will be able to accurately understand the impact of psychological anxiety on menstrual irregularities. This study should be investigated in more detail to determine the physiological and molecular basis for the menstrual irregularities. In addition, this study was compiled mainly from the results of the simple questionnaire (cf: STAI), and it will be necessary in the future to verify the validity of the study results using physiological data, including blood hormone levels.

## 5. Conclusions

Using a multiple logistic model, this study showed that mental anxiety in relatively young elite endurance athletes aged 16–23 years was related to menstrual irregularities.

## Figures and Tables

**Table 1:** Mean values of physical characteristics and state anxiety.

Height (cm)	Body Mass (kg)	%BF (%)	BMI	State Anxiety	Trait Anxiety
168.9 ± 0.3	63.7 ± 0.3	23.8 ± 0.6	22.3 ± 0.1	37.8 ± 0.9	42.1 ± 0.8

BF: body fat, BMI: body mass index.

**Table 2:** Mean values of energy and macronutrient intake.

Energy (kcal)	Protein (g)	Fat (g)	CHO (g)	EA (kcal/FFMkg)
2665.4 ± 47.6	109.2 ± 2.3	87.9 ± 2.1	349.1 ± 7.4	37.1 ± 1.1

CHO: Carbohydrate, EA: Energy Availability.

**Table 3:** Mean values of micronutrient intake.

Ca (mg)	Fe (mg)	VA (µg RAE)	VB_1_ (mg)	VB_2_ (mg)	VC (mg)	Fiber (g)
860.6 ± 34.3	12.4 ± 0.4	1052.0 ± 125.0	1.7 ± 0.1	2.1 ± 0.1	173.4 ± 9.4	16.5 ± 0.5

Ca: calcium, Fe: iron, VA: vitamin A, VB1: vitamin B1, VB2: vitamin B2, VC: vitamin C.

**Table 4:** Variables and estimates of each variable of the multiple logistic models.

Variables	Estimate ± S.E.	z Value	*p* Value	O.R.	S.E. for O.R.
Energy	0.014 ± 0.009	1.550	0.121	1.014	0.009
Ca	0.004 ± 0.002	2.170	0.030	1.004	0.002
VB_2_	−1.346 ± 0.760	−1.769	0.077	0.260	0.103
BM	−0.585 ± 0.278	−2.106	0.035	0.557	0.119
%BF	0.414 ± 0.206	2.007	0.045	1.513	0.257
EA	−0.728 ± 0.425	−1.714	0.087	0.483	0.139
State Anxiety	0.090 ± 0.033	2.755	0.006 *	1.094	0.035

Energy: energy intake, Ca: calcium intake, VB2: vitamin B2 intake, BM: body mass, BF: body fat, EA: energy availability, O.R.: odds ratio, * means statistical significance: *p* < 0.05/7 = 0.0071.

## Data Availability

The data associated with the paper are not publicly available but are available from the corresponding author upon reasonable request.
